# How limited english proficiency impacts patient engagement with telemedicine: a systematic review

**DOI:** 10.1038/s41746-025-02090-3

**Published:** 2025-11-21

**Authors:** Andrea Huang, John Geracitano, Melissa Coffel, Carl Seashore, Saif Khairat

**Affiliations:** 1 Gillings School of Global Public Health, University of North Carolina at Chapel Hill, Chapel Hill, NC USA; 2 School of Nursing, University of North Carolina at Chapel Hill, Chapel Hill, NC USA; 3 Carolina Health Informatics Program, University of North Carolina at Chapel Hill, Chapel Hill, NC USA; 4 General Pediatrics and Adolescent Medicine, School of Medicine, University of North Carolina at Chapel Hill, Chapel Hill, NC USA; 5 Lineberger Comprehensive Cancer Center, University of North Carolina at Chapel Hill, Chapel Hill, NC USA; 6 Cecil G. Sheps Center for Health Services Research, University of North Carolina at Chapel Hill, Chapel Hill, NC USA

**Keywords:** Public health, Health care, Health services

## Abstract

Since the COVID-19 pandemic, telemedicine has become increasingly prevalent in healthcare delivery. While telemedicine holds promise to improve health equity, it may exacerbate pre-existing disparities for patients, including those with limited English proficiency (LEP). We systematically reviewed studies examining telemedicine utilization by LEP and English proficient (EP) patients in the United States. Four databases were searched, and 17 studies were included in this review. Among the seven studies analyzing overall utilization of telemedicine, five reported significantly lower utilization for patients with LEP than those with EP. Similarly, five out of seven studies investigating the utilization of telemedicine over in-person visits found lower telemedicine utilization for LEP patients. All seven studies analyzing telemedicine utilization by modality found significantly lower video use for patients with LEP than those with EP. Future work should address access to technology, digital literacy, healthcare system preparedness, and adequate interpreter accessibility.

## Introduction

Telemedicine in the United States (U.S.) healthcare system has steadily expanded over the past decade and was rapidly adopted during the COVID-19 pandemic^1^. The sudden shift to telemedicine during the pandemic maintained healthcare access to many, reduced travel costs and commuting time, and minimized the risk of infectious disease transmission^2,3^. However, the unprecedented expansion of telemedicine and its associated benefits did not reach patients equally, raising concerns around health disparities^4,5^. Lower telemedicine use has been reported among patients with limited English proficiency (LEP)^6,7^. Disparities related to the digital divide and digital literacy also negatively impacted engagement with digital health^3^.

In 2021, over 8% of the U.S. population, which accounts for approximately 25.7 million individuals, identified as LEP^8^. LEP is defined as individuals speaking a language other than English and also speaking limited English that is less than “very well.”^9^ LEP patients experience existing disparities in healthcare, such as lower access to preventive services, fewer visits to the doctor, and more frequent hospital stays^10^. Such disparities contribute to substantial financial strain on healthcare systems. Patients with LEP frequently incur higher healthcare costs due to increased reliance on emergency departments, extended durations of hospitalization, and elevated rates of readmissions and medical complications when professional interpretation services are unavailable^11^. LEP patients face additional challenges to use digital health tools, such as low health literacy, language barriers, and unreliable access to technology^12^. Without consideration of existing disparities, increased use of telemedicine may unintentionally exacerbate inequities in healthcare access for patients^3,13–15^. The U.S. healthcare system is uniquely shaped by federal and state language access laws, reimbursement policies, and variable infrastructure, making the evaluation of telemedicine disparities for LEP populations both urgent and context-specific.

While individual studies have examined telemedicine use among patients with LEP, most are limited to single sites, vary in methodology, and use different outcome measures, making it difficult to draw broad conclusions. Little is known about which telemedicine modalities are most affected, whether disparities vary by clinical setting, or how interpreter integration influences utilization and quality. Furthermore, the extent to which these disparities have persisted, worsened, or improved in the post-pandemic telemedicine landscape remains unclear. This systematic review synthesized existing U.S.-based evidence to compare telemedicine use between patients with LEP and those with English proficiency (EP), identify key structural and technological barriers, and inform health policies and practices that support equitable telemedicine delivery for linguistically diverse populations. To our knowledge, this is the first systematic review to focus specifically on LEP patients and telemedicine utilization in the U.S.

## Results

### Study selection results

**Fig. 1:**
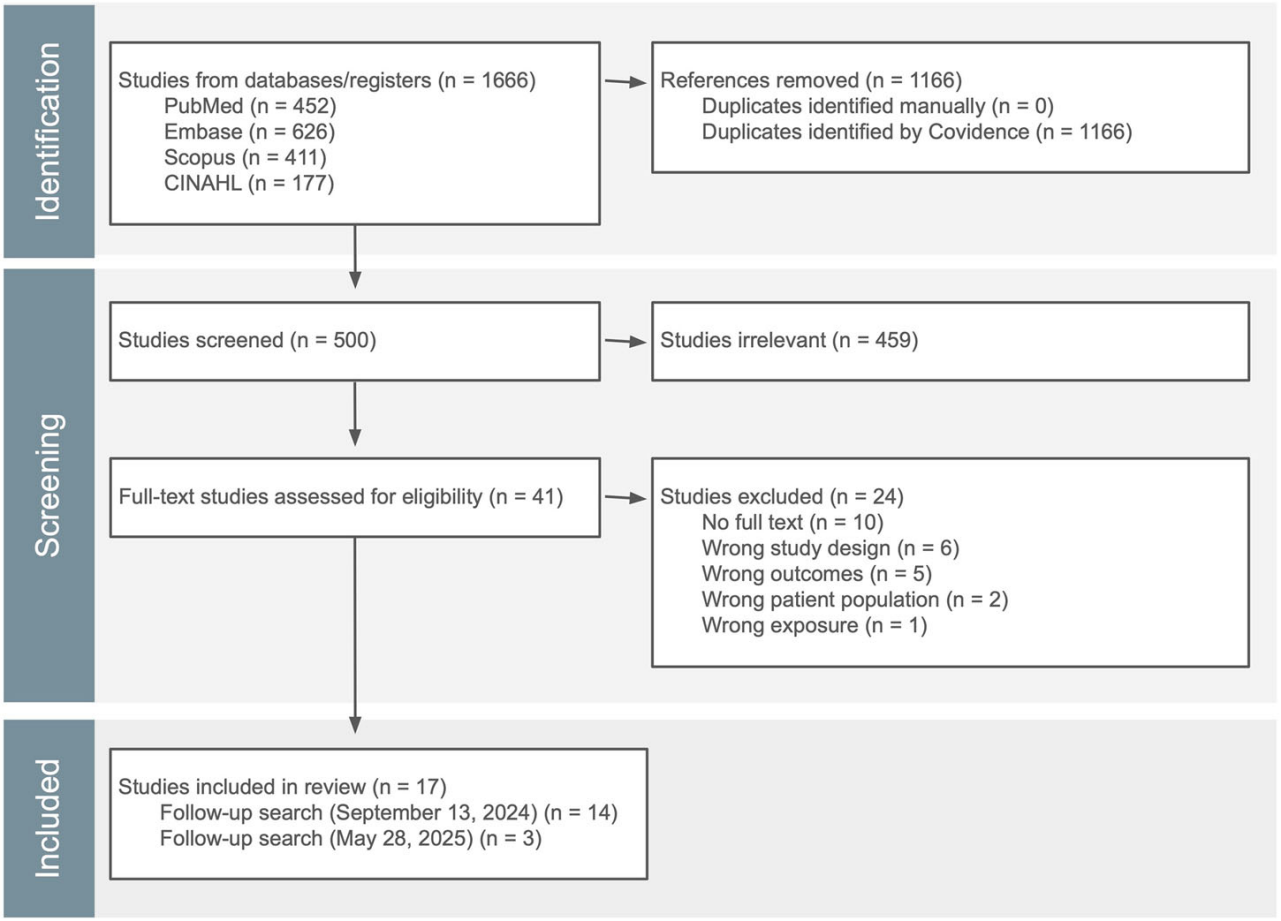
PRISMA flow diagram. This PRISMA flow diagram summarizes the number of records identified through the database search, screened for eligibility, and included in this systematic review.

The initial search on September 13, 2024, yielded 683 publications. To ensure comprehensiveness, the search strategy was expanded to include additional keywords, and a final search was conducted on May 28, 2025, yielding 983 records (Supplementary Table 1). The publications were imported to Covidence for screening, resulting in 500 unique studies after removing duplicates (Fig. 1). Of these, 41 full texts were screened for eligibility, and seventeen studies were ultimately included in this review. A summary of findings for the included studies is presented in Table 1, and Supplementary Table 2 lists the excluded articles along with the reasons for exclusion.

**Table 1:** Summary of Findings

Author (Year)	Data Timeframe	Data Source	Study Design	Location	Specialty	Exposure (LEP) Description	Sample Size	Virtual Visit Description	Primary Outcome	Effect Measure (95% CI, if provided); Reference = EP	Quality
Azizi et al. (2024)^23^	March 1, 2020–December 31, 2022	EHR	Retrospective cohort	California	Oncology	1) Spanish preferred 2) Vietnamese preferred	Spanish: 967 Vietnamese: 461 English: 5951 Other: 511	Telehealth: virtual interaction between provider and patient, including phone calls or video visits	Telehealth utilization over time per month	1) mean difference: −5%, *p* < 0.001 2) mean difference: −5%, *p* < 0.001	3
Chang et al. (2023)^21^	July 2020–December 2021	National Health Interview Survey	Cross-sectional	National (U.S.)	General/ multispecialty	Interview conducted in: Spanish, both English and Spanish, or another language	LEP: 1488 EP: 25,873	Telemedicine visit: appointment with a doctor, nurse, or other health professional by video or by phone	Telemedicine visit in past 12 months	aOR1: 0.56 (0.47–0.67), *p* < 0.001 aOR2: 0.52 (0.44–0.62), *p* < 0.001 aOR3: 0.80 (0.66–0.96), *p* = 0.02	4
Eberly et al. (2020)^34^	March 2020– May 2020	EHR	Retrospective cohort	Pennsylvania and New Jersey	General/ multispecialty	Non-English language as preferred language	LEP: 3895 EP: 144,099 Missing: 408	Telemedicine: visit conducted via video or telephone	Video utilization (vs. telephone-only)	aOR: 0.85 (0.76–0.95)	3
Gordon et al. (2024)^24^	April 2020– December 2020	EHR	Cross-sectional	Greater San Francisco Bay Area, Sacramento area, Silicon Valley, and Central Valley	General/ multispecialty	1) Latino ethnicity with Spanish as preferred language 2) Chinese ethnicity with Chinese dialect as preferred language	LEP Latino: 80,869 EP Latino: 214,765 LEP Chinese: 23,430 EP Chinese: 49,710	Virtual visits conducted by video or phone	At least one video visit during study period	(Non-LEP to LEP ratio): 1) aPR^a^: 1.27 (1.24–1.30) aPR^b^: 1.41 (1.39–1.43) aPR^c^: 1.3 (1.33–1.40) aPR^d^: 1.03 (0.99–1.07) 2) aPR^a^: 1.10 (1.05–1.14) aPR^b^: 1.18 (1.15–1.22) aPR^c^: 1.20 (1.17–1.23) aPR^d^: 1.05 (1.01–1.09)	4
									At least one phone visit during study period	1) aPR^a^: 0.93 (0.92–0.94) aPR^b^: 0.92 (0.91–0.92) aPR^c^: 0.88 (0.87–0.89) aPR^d^: 0.96 (0.95–0.97) 2) aPR^a^: 0.95 (0.92–0.98) aPR^b^: 0.91 (0.90–0.93) aPR^c^: 0.78 (0.76–0.80) aPR^d^: 0.89 (0.87–0.92)	
									Video visits only	1) aPR^a^: 1.43 (1.36–1.50) aPR^b^: 1.78 (1.73–1.83) aPR^c^: 1.85 (1.75–1.96) aPR^d^: 1.28 (1.18–1.40) 2) aPR^a^: 1.11 (1.03–1.20) aPR^b^: 1.25 (1.20–1.31) aPR^c^: 1.47 (1.41–1.54) aPR^d^: 1.29 (1.20–1.39)	
									Phone visits only	1) aPR^a^: 0.82 (0.80–0.83) aPR^b^: 0.79 (0.78–0.80) aPR^c^: 0.75 (0.73–0.76) aPR^d^: 0.98 (0.95–1.01) 2) aPR^a^: 0.88 (0.3–0.93) aPR^b^: 0.84 (0.82–0.86) aPR^c^: 0.69 (0.66–0.72) aPR^d^: 0.92 (0.87–0.98)	
Hsueh et al. (2021)^32^	March 2020– October 2020	EHR	Cross-sectional	California	Primary care	EHR-documented interpreter need	LEP: 22,476 EP: 932,876	Telemedicine: visits conducted via video or telephone	Video utilization (vs. telephone-only)	aOR: 0.77 (0.74–0.80)	4
Jadoo et al. (2023)^29^	2021	EHR	Cross-sectional	Minnesota	Primary care - family medicine pharmacist	Preferred language other than English	LEP visits: 379 EP visits: 436	Virtual visit (by phone or video) (vs. in-person)	Virtual visit (vs. in-person)	absolute difference: –11.2%, *p* < 0.001	4
Meurice et al. (2024)^27^	June 2021–September 2021	EHR	Retrospective cohort	San Diego, Riverside, and Imperial counties in California	Contraceptive care	Spanish-speaking	Spanish: 1274 English: 15,512 Other: 69	Telemedicine: video or audio-only visits	Telemedicine utilization (vs. in-person only)	aOR: 0.53 (0.44–0.64), *p* < 0.001	3
Osmanlliu et al. (2023)^7^	March 2020–February 2021	EHR	Retrospective cohort	Northern California	Cardiology	Non-English language as preferred language	LEP: 7261 EP: 51,427 Missing: 252	Telemedicine: visit delivered by video versus phone	Telemedicine utilization (vs. in-person only)	OR: 0.80 (0.72–0.88)	3
									Video utilization (vs. telephone-only)	OR: 0.52 (0.46–0.59)	
Parameswaran et al. (2023)^30^	March 2020–May 2022	EHR	Retrospective cohort	California Bay Area	Primary care	1) Spanish preferred 2) Mandarin preferred 3) Interpreter usage	New, Non-English: 1072 New, English: 19,959 Return, Non-English: 13,774 Return, English: 193,518 New, Interpreter: 703 New, No Interpreter: 20,319 Return, Interpreter: 10,548 Return, No Interpreter: 196,735	Telemedicine: phone and video visit	Telemedicine visit (vs. in-person) for new patient visits	1) OR: 0.60 (0.44–0.81) 2) OR: 0.74 (0.56–0.98) 3) OR: 1.83 (1.41–1.83)	3
									Telemedicine visit (vs. in-person) for returning patient visits	1) OR: 0.70 (0.63–0.77) 2) OR: 1.04 (0.95–1.14) 3) OR: 1.19 (1.10–1.28)	
Rodriguez et al. (2021)^20^	2015–2018	California Health Interview Survey	Cross-sectional	California	General/ multispecialty	Speak English not well or not at all	LEP: 8063 EP: 76,356	Telehealth: care from a doctor or health professional through a video or telephone conversation rather than an office visit	Telehealth visit in past 12 months	OR: 0.36, *p* < 0.001 aOR: 0.56, *p* < 0.001	4
Rodriguez et al. (2024)^19^	2021	California Health Interview Survey	Cross-sectional	California	General/ multispecialty	Speak English not well or not at all	Video LEP, weighted: 840,764 Video EP, weighted: 11,485,189 Telephone LEP, weighted: 1,021,909 Telephone EP, weighted: 11,524,679	Telehealth: video or telephone visits	Telehealth visit in past 12 months	OR: 0.63 (0.52–0.77), *p* < 0.001	4
Sachs et al. (2021)^26^	June 2020–September 2020	EHR	Cross-sectional	Portland, OR	General/ multispecialty	1) Spanish preferred 2) Other non-English language preferred	Spanish: 3931 Other language: 2156 English: 128,207	Telehealth: video or telephone visit modality	Telehealth utilization (vs. in-person only)	1) aOR: 0.63 (0.59–0.69), *p* < 0.001 2) aOR: 0.76 (0.69–0.3), *p* < 0.001	4
									Video utilization (vs. telephone-only)	1) aOR: 0.20 (0.17–0.23), *p* < 0.001 2) aOR: 0.41 (0.25–0.48), *p* < 0.001	
Thomason et al. (2022)^22^	April 2020–March 2021	EHR	Retrospective cohort	Seattle, Washington	Rheumatology	1) Spanish 2) Other non-English language preferred	Spanish: 174 Non-English/non-Spanish: 147 English: 1121	Telemedicine: video visit	At least one video visit during the study period	1) OR: 0.27 (0.19–0.40), *p* < 0.001 aOR: 0.27 (0.15–0.47), *p* < 0.001 2) OR: 0.23 (0.15–0.36), *p* < 0.001 aOR: 0.32 (0.19–0.53), *p* < 0.001	3
Tong et al. (2022)^28^	March 2020–March 2022	EHR	Retrospective cohort	Wisconsin	Oncology	Interpreter required	LEP: 660 EP: 45,805	Telemedicine: video-based telemedical visit	Telemedicine utilization (vs. in-person only)	OR: 0.49 (0.39–0.6), *p* < 0.001 aOR: 0.4 (0.30–0.52), *p* < 0.001	3
Wakeman et al. (2025)^25^	February 23, 2022– August 26, 2022	Online survey	Cross-sectional	National (U.S.)	General/ multispecialty	Self-reported proficiency of less than very well (well, not well, not at all)	LEP: 460 EP: 4984	Telehealth: video or phone visit (not including emergency room visits)	Telehealth in the past 12 months	aOR: 1.54 (1.23–1.92)	4
								Telemedicine: remote counseling or remotely supervised training or therapy from a healthcare [clinician] by video or phone in the past 12 months	Telemedicine in the past 12 months	aOR: 1.60 (1.28–1.99)	
Yoon et al. (2024)^33^	December 2020 and March 2021	Survey	Cross-sectional	Chicago, IL	Primary care	Limited English proficiency (no additional description provided)	LEP: 61 EP: 657	Telehealth/telemedicine: appointment visit via video or telephone	Video modality as most recent telemedicine visit (vs. telephone-only)	OR: 0.36 (0.13–0.95), *p* ≤ 0.05	4
Zachrison et al. (2021)^31^	October 1, 2019– September 30, 2020 (COVID data subset: March 2020–September 2020)	EHR	Retrospective cohort	Northeast U.S.	General/ multispecialty	Non-English language as preferred language	LEP: 101,899 English: 1,128,785 Missing: 10,629 COVID LEP: 65,710 COVID English: 789,908 COVID Missing: 4483	Virtual visit: virtual video or audio visits	Telemedicine utilization (vs. in-person only)	OR: 1.02 (1.01–1.04) aOR: 1.034 (1.01–1.06)	3
									Telemedicine utilization (vs. in-person only) – COVID-19	OR: 1.08 (1.06–1.11) aOR 1.05 (1.03–1.08)	
									Video utilization (vs. telephone —only)— COVID-19	OR: 0.57 (0.55–0.58) aOR: 0.89 (0.86–0.93)	

*LEP* limited English proficiency, *EP* English proficiency, *EHR* Electronic Health Records, *OR* odds ratio, *aOR* adjusted odds ratio, *aPR* adjusted. prevalence ratio.

^a^Age group: 26–39 y.

^b^Age group: 40–64 y.

^c^Age group: 65–75y.

^d^Age group: 76–85 y.

### Quality assessment

Using the modified Oxford Centre for Evidence-based Medicine (OCEBM) rating scheme, eight of the 17 studies in this review have a quality rating of three, and nine have ratings of four (Table 1), reflecting their retrospective cohort and cross-sectional study designs^16,17^. The Grading of Recommendations, Assessment, Development, and Evaluations (GRADE) method showed that quality was low to moderate overall (Table 2), mainly due to the observational nature of these studies as well as inconsistencies in what population characteristics the studies used to proxy LEP^18^.

**Table 2:** GRADE Quality Assessment

GRADE Evidence Profile: Virtual Care Engagement for Patients with LEP						
Quality Assessment						
No. of Studies (Design)	Limitations	Inconsistency	Indirectness	Imprecision	Publication Bias	Quality
Outcome #1: Telemedicine visit in the past 12 months / in study period						
5 (Cross Sectional)	Serious limitations (because of varying definitions of LEP)	Serious inconsistency (inconsistent effects reported)	Serious indirectness (because indirectness of outcome)	No serious imprecision	Undetected	⊕◯◯◯Low
2 (Retrospective Cohort)	Serious limitations (because of varying definitions of LEP)	No serious inconsistency	No serious indirectness	No serious imprecision	Undetected	⊕⊕◯◯Moderate
Outcome #2: Telemedicine utilization (vs. in-person only)						
2 (Cross Sectional)	Serious limitations (because of varying definitions of LEP)	No serious inconsistency	No serious indirectness	No serious imprecision	Undetected	⊕⊕◯◯Moderate
5 (Retrospective Cohort)	Serious limitations (because of varying definitions of LEP)	Serious inconsistency (opposing effects reported)	No serious indirectness	No serious imprecision	Undetected	⊕◯◯◯Low
Outcome #3: Video utilization (vs. telephone-only)						
4 (Cross Sectional)	Serious limitations (because of varying definitions of LEP)	No serious inconsistency	No serious indirectness	No serious imprecision	Undetected	⊕⊕◯◯ Moderate
3 (Retrospective Cohort)	Serious limitations (because of varying definitions of LEP)	No serious inconsistency	No serious indirectness	No serious imprecision	Undetected	⊕⊕◯◯Moderate

### Study characteristics

Among the 17 studies included, nine used a cross-sectional study design and eight employed a retrospective cohort study design. Sample sizes ranged from *n* = 718 to *n* = 1,241,313. Studies were conducted in the northeast U.S. (*n* = 2), California (*n* = 8), Oregon (*n* = 1), Washington (*n* = 1), Wisconsin (*n* = 1), Illinois (*n* = 1), Minnesota (*n* = 1), and nationally (*n* = 2). Five studies used survey data, and 12 used electronic health records. Studies also spanned different fields of medicine, including primary care (*n* = 4), contraceptive care (*n* = 1), cardiology (*n* = 1), rheumatology (*n* = 1), oncology (*n* = 2), and general/multispecialty (*n* = 8). All studies looked at the ambulatory setting.

These 17 studies were grouped into three review categories of telemedicine use for patients with LEP: 1) overall utilization of telemedicine, 2) utilization of telemedicine compared to in-person visits, and 3) utilization of video- versus telephone-based telemedicine. Among the included studies, 41% (7/17) reported on telemedicine utilization frequency in the last 12 months or during the study period, 41% (7/17) reported on telemedicine utilization compared to in-person visits, and 41% (7/17) reported on video utilization compared to telephone use for telemedicine visits. Three studies reported on more than one of the three categories.

### Outcome 1: Overall telemedicine utilization

Even though telemedicine has emerged as a critical component of healthcare delivery, disparities in its access and utilization persist, particularly among patients with LEP. Five of the seven studies reviewed in this section found significantly lower telemedicine utilization for patients with LEP compared to those with EP^19–23^. One study showed no difference in telemedicine utilization between two populations^24^. One study showed significantly higher telemedicine utilization for patients with LEP^25^.

Three survey studies investigating various care settings found lower telemedicine usage by LEP patients than by EP patients. Using National Health Interview Surveys data on nonelderly adults, Chang et al. reported a significantly lower percentage of patients with LEP having any telemedicine visits compared to those with EP^21^. After adjusting for enabling, predisposing, and need factors, LEP patients had 20% lower odds of having a telemedicine visit compared to EP patients (aOR, 0.80; 95% CI, 0.66–0.96; *p* = 0.02)^21^. Analysis of the 2015-2018 California Health Interview Survey (CHIS) data also found lower rates of telemedicine use by patients with LEP compared to EP speakers (4.8% vs. 12.3%)^20^. A later study using the 2021 CHIS adult data similarly found that patients with LEP were less likely to report using telemedicine (video or telephone) compared to patients with EP (OR, 0.63; 95% CI, 0.52–0.77; *p* < 0.001)^19^.

A similar negative association was seen in a rheumatology setting in Washington state. Thomason et al. reported that patients who preferred Spanish or other non-English languages were less likely to use video-based visits than patients who preferred English (Spanish OR, 0.27; 95% CI, 0.15–0.47; *p* < 0.001; other non-English languages OR, 0.34; 95% CI, 0.21–0.55; *p* < 0.001)^22^.

Lower telemedicine usage by LEP patients compared to EP patients was seen in oncology, as well as in a study looking at patients in a California cancer center. Azizi et al. found that patients with Spanish- and Vietnamese-preferences were both less likely to have had virtual visits than EP patients (Spanish: mean difference: −5%, *p* < 0.001; Vietnamese: mean difference: −5%, *p* < 0.001)^23^.

In contrast to the statistically significant lower LEP patient telemedicine use in the studies above, one study analyzing telemedicine utilization stratified ethnic groups by Chinese and Latino populations in California and found no significant difference between LEP and EP groups. This study, conducted by Gordon et al., examined patients with at least one outpatient visit in the first nine months of the COVID-19 pandemic and reported a similar prevalence of virtual care use between LEP and EP patients for both Chinese and Latino groups^24^.

One cross-sectional study conducted via survey, looking at counties across the U.S. falling under the highest and lowest quartiles of the Minority Health Social Vulnerability Index, reported that those with LEP had higher adjusted odds of utilizing telemedicine^25^. Patients with LEP in these quartiles had 1.6 times the odds of non-emergency room-related video or phone visits compared to those with EP (aOR, 1.60; 95% CI, 1.28–1.99)^25^.

### Outcome 2: Telemedicine utilization compared to in-person visits

In addition to the studies investigating overall telemedicine utilization, seven studies directly compared telemedicine use to in-person visits. Five of the seven studies reported lower odds of telemedicine utilization for LEP patients^7,26–29^. One study reported opposite patterns of telemedicine utilization depending on interpreter use^30^, while another observed slightly higher telemedicine usage among LEP patients^31^.

For ambulatory clinics in Oregon, Sachs et al. found that patients who preferred Spanish or other non-English languages had lower odds of using telemedicine compared to EP individuals (Spanish OR, 0.63; 95% CI, 0.59–0.69; *p* < 0.001; Other non-English OR, 0.20; 95% CI, 0.17–0.23; *p* < 0.001)^26^. Similar negative associations between telemedicine utilization and LEP were reported by Osmanlliu et al. in California. For adult cardiovascular ambulatory care, patients with non-English language preferences had lower odds of telemedicine visits (OR, 0.80; 95% CI, 0.72–0.88)^7^. A study on contraceptive healthcare services in California by Meurice et al. also showed that patients with Spanish language preference had significantly lower adjusted odds of choosing telemedicine over in-person care (OR, 0.53; 95% CI, 0.44–0.64; *p* < 0.001)^27^.

Similarly, a lower utilization of virtual visits vs. in-person visits by patients with LEP compared to EP was reported for clinical pharmacist visits in a family medicine clinic in Minnesota^29^. LEP patients had 11.2% fewer virtual visits compared to EP patients (absolute difference: −11.2%; *p* < 0.001)^29^.

A study by Tong et al. in an oncology facility in Wisconsin found that patients needing interpreter services had significantly lower odds of using telemedicine compared to in-person visits (OR, 0.4; 95% CI, 0.30–0.52; *p* < 0.001)^28^. Unlike previous studies, which used language preference to classify LEP or EP, this study defined patients with LEP as those documented as requiring an interpreter.

While the previous studies showed consistently lower telemedicine utilization compared to in-person visits for patients with LEP, one study by Parameswaran et al. reported an opposite association of telemedicine utilization when looking at LEP defined by either language preference or interpreter need^30^. This study, conducted at a large academic medical center in California, investigated primary care telemedicine visits for new and returning patient visits. For new patient visits with patients with Spanish and Mandarin language preferences, there was a significantly lower utilization of telemedicine compared to in-person visits for both language groups (Spanish OR, 0.60; 95% CI, 0.44–0.81; Mandarin OR, 0.74; 95% CI, 0.56–0.98). For returning patient visits, a significantly lower telemedicine utilization was seen only for Spanish preference (Spanish OR, 0.70; 95% CI, 0.63–0.77; Mandarin OR, 1.04; 95% CI, 0.95–1.14). However, when looking at interpreter service use rather than language preference, a significantly greater utilization of telemedicine was found for both new (OR, 1.83; 95% CI, 1.41–2.38) and returning patient visits (OR, 1.19; 95% CI, 1.10–1.28)^30^.

In contrast to most of the other studies, one study found that patients with non-English preference had slightly higher adjusted odds of using telemedicine over in-person participation^31^. Zachrison et al. examined ambulatory care visits in a large New England healthcare system and found slightly higher adjusted odds for patients with a non-English language preference (aOR, 1.034; 95% CI, 1.01–1.06; COVID-19 timeframe: aOR, 1.05; 95% CI, 1.03–1.08). This study noted that the different trend may have reflected, at least partially, their pre-pandemic adoption of telemedicine and better health system preparedness^31^.

### Outcome 3: Video compared to telephone use for telemedicine

Seven studies investigated differences in telemedicine utilization by modality, video versus telephone, among patients with LEP. All the studies showed significantly less video use by patients with LEP compared to patients with EP^7,24,26,31–34^. One study reported that while LEP was associated with lower use of video than audio, for the subset of patients with prior experience with video visits, there was no difference in visit modality between patients with LEP compared to EP^32^.

A study by Yoon et al. involving patients with chronic conditions at higher risk for COVID-19 from a group of academic and community health centers in Illinois showed that patients with LEP had significantly lower odds of using video for telemedicine visits compared to those with EP (OR, 0.36; 95% CI, 0.13–0.95)^33^. Similarly, a large academic health system in the Pennsylvania and New Jersey area found that LEP patients had lower adjusted odds of utilizing video for telemedicine visits in the primary care setting (OR, 0.85; 95% CI, 0.76–0.95)^34^. A similar negative association was seen for LEP video visits for primary and specialty ambulatory care at another Northeast hospital system (OR: 0.89; 95% CI, 0.86–0.93)^31^. A study by Osmanlliu et al. in a cardiovascular ambulatory care setting in Northern California found that LEP patients were less likely to use video visits compared to telephone visits (OR, 0.52; 95% CI, 0.46–0.59)^7^.

Sachs et al.’s study at ambulatory clinics in Oregon looked at patients who preferred Spanish and other non-English languages. Both LEP groups had lower odds of using video visits than EP patients, with a stronger association for those preferring Spanish (Spanish OR, 0.20; 95% CI, 0.17–0.23; *p* < 0.001; other non-English OR, 0.41; 95% CI, 0.25–0.48; *p* < 0.001)^26^.

Gordon et al.’s study on primary and specialty healthcare in California looked at two ethnic groups and compared LEP and EP patients within each group. It was also concluded that, within the same age strata, LEP adults were more likely to use phone visits and less likely to use video visits than EP adults for both Latino and Chinese populations^24^.

Studies that defined LEP as the need for interpreters reported a similar negative association. Hsueh et al.’s study on primary care visits in California found that LEP individuals were significantly less likely to use video-based visits compared to EP patients, and often defaulted to telephone-only consultations (OR, 0.77; 95% CI, 0.74–0.80)^32^. However, further analysis showed that among patients with prior video visit experience, there was not a significant difference between LEP patients’ and EP patients’ likelihood of choosing the video visit modality (47.2% vs 49.1%; *p* = 0.09).

## Discussion

As the prevalence of telemedicine is increasing, it is crucial to ensure that other vulnerable populations, including those with LEP, are not left behind^2,3^. In this review, we identified disparities in telemedicine usage between LEP and EP patients. Overall, patients with LEP were less likely to have used telemedicine and also less likely to have utilized telemedicine over in-person healthcare. Moreover, LEP patients were less likely to utilize video over audio modalities for telemedicine. These differences highlight the importance of addressing telemedicine access and delivery for LEP patients.

Lower utilization of telemedicine by LEP patients was seen across various healthcare settings, including primary and specialty ambulatory care settings, such as rheumatology^22^, contraceptive care^27^, cardiology^7^, and oncology^28^. Studies included in this review suggested that factors such as lack of equipment, low digital literacy, limited interpreter availability, and insufficient language-concordant care contributed to these observations^7,19–22,26,27,30^. Qualitative research through provider interviews cited additional barriers for LEP patients, including private space, age, and personal preference as reasons for not using telemedicine^35^. Another qualitative study of telemedicine among multilingual patients points out concerns about managing their health without in-person physical evaluation^36^. The lower telemedicine utilization found in this review is similar to the trends of overall lower healthcare access and utilization by LEP patients^37,38^.

In addition to underutilization, there are some disparities in LEP patients’ experience interacting with virtual care. For example, Rodriguez reported that LEP patients had worse experiences with video visits than in-person care, which may contribute to their reluctance to adopt video-based telemedicine^19^. Encouraging virtual healthcare use among those not comfortable with the technology may lead to inefficient visits, decreased satisfaction, and potential overutilization of emergency departments and extended hospital stays, leading to increased healthcare costs^39–41^.

Less video use compared to the audio-only modality by patients with LEP was observed consistently in all included studies. Differences between video and telephone visits for LEP patients provide valuable insights into their needs and preferences, which can further help to improve virtual care for this population. Telephone visits are more accessible and play an important role in maintaining healthcare access to groups like LEP patients^28,31^. However, video visits provide higher-quality, more interactive, and more effective healthcare experiences^31^. Video visits allow for enhanced patient-provider interaction through visual assessments and non-verbal communication^32^. The utilization of video modality is particularly important for specialties, such as cardiovascular care, in which visual assessments benefit the management of disease conditions^7^. Research also suggests that video visits are associated with better diagnostic accuracy, improved decision-making, and fewer medication errors than telephone visits^42^.

Several factors influence patient choice between video and telephone visits, including lack of internet access, video-compatible devices, and digital literacy^26,31,32,34^ Additional layers of difficulty faced by LEP patients include limited interpreters available for video visits, unfamiliarity with video-based healthcare platforms, and inadequate language support^26,34^. Hsueh et al.’s finding of no significant difference in choosing video visits between LEP and EP patients who have prior video visits also suggests the importance of breaking the initial barriers for LEP patients for video use^32^. Altogether, this suggests that virtual healthcare through a video modality has the potential to improve satisfaction and health outcomes, but patients with LEP may not be able to benefit completely from this modality due to these various challenges.

Definitions of limited English proficiency varied across the included studies. Most studies used non-English language preference, some distinguished between Spanish and other non-English languages, and others used proxies such as a documented need for an interpreter. Interestingly, telemedicine utilization varied when LEP was measured by the need for an interpreter^30^. Limited interpretation service availability also restricted one of the studies to offering phone-based telemedicine without the option for video for their non-English patients^27^. These underscore discrepancies in LEP measurement and potential implications for interpreter availability and adequate language support^43^. Using professional interpreters can alleviate the burden on providers to rely on their own potentially limited language skills or use ad hoc interpreters, practices that may contribute to poorer health outcomes^44,45^. To address this, training and clearer guidelines for providers on the decision to call a medical interpreter could better prepare them to engage with patients^43^. Assessments on the adequacy of interpreter services for telemedicine could help determine the capacity of healthcare systems to support patients with LEP. Additionally, with the advancement of technology, integrating multiple users in virtual visit platforms for incorporating services^46,47^ and evaluating if digital navigators improve the video visit experience could be explored^19^. Additional research could look at the effectiveness of ambient listening with real-time automated translation compared to professional interpreters, which might eliminate the need for an interpreter to be present in the virtual visits^48–50^.

Furthermore, establishing a clear definition of LEP could help ensure that studies are not leaving behind other individuals who experience disparities in healthcare access due to language barriers. The terminology “LEP” is also controversial, as it centers around the English language and focuses on a limitation or deficiency in English proficiency, which has negative connotations^43^. Instead, some have shifted to using the term “Language other than English” and “Non-English Language Preferred”^29,51^.

There are a few limitations to this systematic review that should be noted. One limitation was that the majority of the included studies were conducted during the COVID-19 pandemic. The sudden and unexpected shift to virtual delivery of healthcare, emergency regulatory changes, insurance reimbursement rules, and public health recommendations are specific to that time period. While telemedicine will continue to be an integral component of healthcare delivery, this overlap limits generalizability to the post-pandemic healthcare landscape. Additionally, this review focused solely on the U.S., so findings may not be generalizable to other countries with different telemedicine policies and healthcare systems. Many studies also focused on specific geographic locations and hospital systems, so generalizability to other settings may be limited. Other aspects of remote communication for healthcare, such as accessing patient portals, messaging over applications, emailing, and telemonitoring, were not investigated in this review. Grey literature, such as conference presentations and abstracts, was not included. Grey literature can be more current and present ideas that highlight emerging trends, and including these unpublished results could affect publication bias. Lastly, there was a small number of studies included in this review, and no meta-analysis was conducted, which was out of the scope of the research question. As a result, formal assessments of heterogeneity and pooled estimates of association were not generated. Additionally, the low to moderate overall quality assessment limits the confidence in conclusions drawn from the review.

In addition to the limitations of this review, there are also some limitations with the studies included. All studies relied on surveys or electronic health records as the data source, and the data quality can be hard to control due to potential inconsistencies or inaccuracies in documentation. Studies relied on surveys and self-reported utilization of services, which can be affected by recall bias and have variable accuracy^52^. Additionally, some studies acknowledge unaccounted factors, including patient, clinician, and institutional level factors that could contribute to the preference for telemedicine use^19,20,26,30^.

Virtual healthcare has become more integrated in the U.S. healthcare system, particularly since the COVID-19 pandemic, and has played a role in improving healthcare accessibility. As telemedicine continues to advance and transform healthcare delivery, steps need to be taken to ensure that populations, including patients with LEP, are not left behind. Understanding how LEP patients engage with different telemedicine modalities and addressing specific barriers to telemedicine can help create a more equitable virtual healthcare system. Future research may explore targeted interventions to enhance telemedicine platform designs to ensure accessibility, improve digital literacy, and ensure access to robust interpreter services to improve LEP patients’ experience.

## Methods

This systematic review was conducted following the guidelines for Preferred Reporting Items for Systematic Reviews and Meta-analyses (PRISMA)^53^.

### Search strategy

The databases Cumulative Index of Nursing and Allied Health Literature (CINAHL), EMBASE, PubMed, and Scopus were searched on May 28, 2025. Our search strategy included search terms with Limited English Proficiency, telemedicine, and equivalent words (Supplementary Table 1).

### Inclusion criteria

Peer-reviewed studies conducted in the U.S. and published in English between January 2019 and May 2025 were included. Eligible study designs included randomized controlled trials, cohort studies, case-control studies, and cross-sectional studies. Only adult patients (age 18 years and older) were included, and studies must clearly define LEP as an exposure and stratify results by English proficiency.

### Exclusion criteria

Studies were excluded if they were conducted outside of the U.S. or in a non-English language due to differences in healthcare systems. Studies without LEP-stratified outcomes or no telemedicine utilization outcomes were also excluded. Grey literature, opinion pieces, editorials, systematic reviews, qualitative case studies, and abstract-only publications were not included. Studies evaluating specific programs/interventions, including feasibility and pilot studies, were also excluded.

### Study selection process

Database searches of publications between January 2019 and May 2025 were included in this review. All results were uploaded to Covidence, an online screening software, and duplicates were removed. Two reviewers (AH and JG) screened titles and abstracts following the eligibility criteria. Conflicts were resolved through consensus. Two reviewers (AH and JG) conducted full-text screening following the same criteria, with conflicts resolved through consensus. Manual data extraction was conducted by one reviewer (AH). Data extracted included the population age group of interest, medical specialty, location of data collection, sample size, period of data collection, study design, exposure, primary outcomes, findings, and LEP as defined by each study. The quality of evidence was assessed following a modified rating scheme from the OCEBM and the GRADE system^16–18^. Risk of bias was not formally assessed for included studies due to their observational nature.

### Quality assessment

The quality of individual studies was assessed using the modified OCEBM rating scheme (Supplementary Table 3), with ratings of 1–5, where 1 is defined as properly powered and having a randomized clinical trial design or a systematic review including meta-analysis, while 5 is defined as the opinion of respected authorities or a case report. Two reviewers (AH and JG) performed quality assessment, and any conflicts were resolved through consensus.

## Supplementary information

Supplementary information

## Data Availability

The data extracted for this study are available from the corresponding author upon reasonable request.

## References

[1] Shaver, J. The State of Telehealth Before and After the COVID-19 Pandemic. *Prim. Care* **49**, 517–530 (2022). 36357058 10.1016/j.pop.2022.04.002 PMC9035352

[2] Johns Hopkins Medicine. Benefits of Telemedicine. *Johns Hopkins Medicine*. https://www.hopkinsmedicine.org/health/treatment-tests-and-therapies/benefits-of-telemedicine (2022).

[3] Gergen Barnett, K. et al. Telehealth’s Double-Edged Sword: Bridging or Perpetuating Health Inequities?. *J. Gen. Intern. Med.* **37**, 2845–2848 (2022). 35352272 10.1007/s11606-022-07481-w PMC8963395

[4] World Health Organization. Health equity. https://www.who.int/health-topics/health-equity.

[5] Office of Disease Prevention and Health Promotion. Health Equity in Healthy People 2030 - Healthy People 2030. https://health.gov/healthypeople/priority-areas/health-equity-healthy-people-2030.

[6] Luo, J. et al. Telemedicine Adoption during the COVID-19 Pandemic: Gaps and Inequalities. *Appl. Clin. Inform.* **12**, 836–844 (2021). 34496419 10.1055/s-0041-1733848 PMC8426040

[7] Osmanlliu, E. et al. Sociodemographic disparities in the use of cardiovascular ambulatory care and telemedicine during the COVID-19 pandemic. *Am. Heart J.* **263**, 169–176 (2023). 37369269 10.1016/j.ahj.2023.06.011 PMC10290766

[8] Haldar, S., Pillai, D. & Published, S. A. Overview of Health Coverage and Care for Individuals with Limited English Proficiency (LEP). *KFF* https://www.kff.org/racial-equity-and-health-policy/issue-brief/overview-of-health-coverage-and-care-for-individuals-with-limited-english-proficiency/ (2023).

[9] LEP.gov. Source and Methodology. https://www.lep.gov/source-and-methodology (2020).

[10] Eberly, L. A. et al. Telemedicine Outpatient Cardiovascular Care During the COVID-19 Pandemic. *Circulation* **142**, 510–512 (2020). 32510987 10.1161/CIRCULATIONAHA.120.048185 PMC9126131

[11] Quick Safety 13: Overcoming the challenges of providing care to limited English proficient patients | The Joint Commission. https://www.jointcommission.org/resources/news-and-multimedia/newsletters/newsletters/quick-safety/quick-safety--issue-13-overcoming-the-challenges-of-providing-care-to-lep-patients/overcoming-the-challenges-of-providing-care-to-lep-patients/.

[12] Philpot, L. M., Ramar, P., Roellinger, D. L., McIntee, M. A. & Ebbert, J. O. Digital health literacy and use of patient portals among Spanish-preferred patients in the United States: a cross-sectional assessment. *Front. Public Health* **12**, 1455395 (2024). 39720810 10.3389/fpubh.2024.1455395 PMC11666482

[13] Callisaya, M. L., Lee, A. H.-C. & Khushu, A. Rapid implementation of telehealth in geriatric outpatient clinics due to COVID-19. *Intern. Med. J.* **51**, 1151–1155 (2021). 34143563 10.1111/imj.15306 PMC8444941

[14] Ye, S. et al. Telemedicine Expansion During the COVID-19 Pandemic and the Potential for Technology-Driven Disparities. *J. Gen. Intern. Med.* **36**, 256–258 (2021). 33105000 10.1007/s11606-020-06322-y PMC7586868

[15] Bakhtiar, M., Elbuluk, N. & Lipoff, J. B. The digital divide: How COVID-19’s telemedicine expansion could exacerbate disparities. *J. Am. Acad. Dermatol.* **83**, e345–e346 (2020). 32682890 10.1016/j.jaad.2020.07.043 PMC7365110

[16] JAMA. Instructions for Authors: Ratings of the quality of the evidence.

[17] OCEBM Levels of Evidence Working Group. The Oxford Levels of Evidence 2. *Oxford Centre for Evidence-Based MedicineOxford Centre for Evidence-Based Medicine* https://www.cebm.ox.ac.uk/resources/levels-of-evidence/ocebm-levels-of-evidence.

[18] Guyatt, G. et al. GRADE guidelines: 1. Introduction—GRADE evidence profiles and summary of findings tables. *J. Clin. Epidemiol.* **64**, 383–394 (2011). 21195583 10.1016/j.jclinepi.2010.04.026

[19] Rodriguez, J. A. et al. Telehealth Experience Among Patients With Limited English Proficiency. *JAMA Netw. Open* **7**, e2410691 (2024). 38722633 10.1001/jamanetworkopen.2024.10691 PMC11082683

[20] Rodriguez, J. A., Saadi, A., Schwamm, L. H., Bates, D. W. & Samal, L. Disparities In Telehealth Use Among California Patients With Limited English Proficiency. *Health Aff. Proj. Hope* **40**, 487–495 (2021). 10.1377/hlthaff.2020.00823 33646862

[21] Chang, E., Davis, T. L. & Berkman, N. D. Differences in Telemedicine, Emergency Department, and Hospital Utilization Among Nonelderly Adults with Limited English Proficiency Post-COVID-19 Pandemic: a Cross-Sectional Analysis. *J. Gen. Intern. Med.* **38**, 3490–3498 (2023). 37592119 10.1007/s11606-023-08353-7 PMC10713935

[22] Thomason, J. et al. Non-English Language Preference Associated With Decreased Rheumatology Telehealth Use During the COVID-19 Pandemic. *ACR Open Rheumatol.* **4**, 385–394 (2022). 35084116 10.1002/acr2.11407 PMC9096515

[23] Azizi, A. et al. Associations between language, telehealth, and clinical outcomes in patients with cancer during the COVID-19 pandemic. *Cancer Med* **13**, e70099 (2024). 39312904 10.1002/cam4.70099 PMC11419674

[24] Gordon, N. P., Lin, T. Y., Torreblanca, A. & Reed, M. E. Video and phone visit use differed by language preference among U.S. Latino and Chinese adults during the first 9 months of the COVID-19 pandemic: a cross-sectional electronic health record study. *BMC Health Serv. Res.* **24**, 900 (2024). 39113055 10.1186/s12913-024-11356-7 PMC11304802

[25] Wakeman, M., Buckman, D. W. & El-Toukhy, S. Disparities in Digital Health Care Use in 2022. *JAMA Netw. Open* **8**, e255359 (2025). 40244585 10.1001/jamanetworkopen.2025.5359 PMC12550827

[26] Sachs, J. W., Graven, P., Gold, J. A. & Kassakian, S. Z. Disparities in telephone and video telehealth engagement during the COVID-19 pandemic. *JAMIA Open* **4**, ooab056 (2021). 34632322 10.1093/jamiaopen/ooab056 PMC8496485

[27] Meurice, M. E., Mody, S. K., Nodora, J., Marengo, A. & Averbach, S. Social determinants of choosing telemedicine for contraceptive care: A retrospective cohort study. *Contraception* **134**, 110414 (2024). 38431258 10.1016/j.contraception.2024.110414

[28] Tong, L. et al. Telemedicine and health disparities: Association between patient characteristics and telemedicine, in-person, telephone and message-based care during the COVID-19 pandemic. *IPEM-Transl.* **3**, 100010 (2022). 36340828 10.1016/j.ipemt.2022.100010 PMC9617798

[29] Jadoo, H., Klemenhagen, K. C., Freeman, K. & Philbrick, A. M. Comparison of clinical pharmacist visits based on language preference in an urban underserved clinic: A cross-sectional analysis. *JACCP J. Am. Coll. Clin. Pharm.* **6**, 1210–1215 (2023).

[30] Parameswaran, V. et al. Drivers of telemedicine in primary care clinics at a large academic medical centre. *J. Telemed. Telecare* 1357633X231219311 10.1177/1357633X231219311 (2023). 10.1177/1357633X231219311 38130140

[31] Zachrison, K. S. et al. Patient characteristics associated with the successful transition to virtual care: Lessons learned from the first million patients. *J. Telemed. Telecare* **29**, 621–631 (2023). 34120506 10.1177/1357633X211015547

[32] Hsueh, L. et al. Disparities in Use of Video Telemedicine Among Patients With Limited English Proficiency During the COVID-19 Pandemic. *JAMA Netw. Open* **4**, e2133129 (2021). 34735015 10.1001/jamanetworkopen.2021.33129 PMC8569485

[33] Yoon, E. et al. Patient factors associated with telehealth quality and experience among adults with chronic conditions. *JAMIA Open* **7**, ooae026 (2024). 38596698 10.1093/jamiaopen/ooae026 PMC11000823

[34] Eberly, L. A. et al. Patient Characteristics Associated With Telemedicine Access for Primary and Specialty Ambulatory Care During the COVID-19 Pandemic. *JAMA Netw. Open* **3**, e2031640 (2020). 33372974 10.1001/jamanetworkopen.2020.31640 PMC7772717

[35] Lu, J. & Bollinger, L. Navigating Telehealth in Limited English Proficiency Populations. *EthnoMed*. https://ethnomed.org/resource/navigating-telehealth-in-limited-english-proficiency-populations/ (2020).

[36] Nguyen, M.-L. T. et al. Satisfaction can co-exist with hesitation: qualitative analysis of acceptability of telemedicine among multi-lingual patients in a safety-net healthcare system during the COVID-19 pandemic. *BMC Health Serv. Res.* **22**, 195 (2022). 35164746 10.1186/s12913-022-07547-9 PMC8842908

[37] Twersky, S. E., Jefferson, R., Garcia-Ortiz, L., Williams, E. & Pina, C. The Impact of Limited English Proficiency on Healthcare Access and Outcomes in the U.S.: A Scoping Review. *Healthcare* **12**, 364 (2024). 38338249 10.3390/healthcare12030364 PMC10855368

[38] Ramirez, N. et al. Access to Care Among Adults with Limited English Proficiency. *J. Gen. Intern. Med.* **38**, 592–599 (2023). 35882706 10.1007/s11606-022-07690-3 PMC9971409

[39] Linkous, J. Challenges in Telehealth. in *The Role of Telehealth in an Evolving Health Care Environment: Workshop Summary* (National Academies Press (US), 2012). 24901186

[40] Morgan, D. J., Leppin, A. L., Smith, C. D. & Korenstein, D. A Practical Framework for Understanding and Reducing Medical Overuse: Conceptualizing Overuse Through the Patient-Clinician Interaction. *J. Hosp. Med.* **12**, 346–351 (2017). 28459906 10.12788/jhm.2738 PMC5570540

[41] Shah, V. V. et al. Association Between In-Person vs Telehealth Follow-up and Rates of Repeated Hospital Visits Among Patients Seen in the Emergency Department. *JAMA Netw. Open* **5**, e2237783 (2022). 36282505 10.1001/jamanetworkopen.2022.37783 PMC9597390

[42] Rush, K. L., Howlett, L., Munro, A. & Burton, L. Videoconference compared to telephone in healthcare delivery: A systematic review. *Int. J. Med. Inf.* **118**, 44–53 (2018). 10.1016/j.ijmedinf.2018.07.007 30153920

[43] Ortega, P., Shin, T. M. & Martínez, G. A. Rethinking the Term “Limited English Proficiency” to Improve Language-Appropriate Healthcare for All. *J. Immigr. Minor. Health* **24**, 799 (2021). 34328602 10.1007/s10903-021-01257-w PMC8323079

[44] Andres, E., Wynia, M., Regenstein, M. & Maul, L. Should I Call an Interpreter?—How do Physicians with Second Language Skills Decide?. *J. Health Care Poor Underserved* **24**, 525–539 (2013). 23728026 10.1353/hpu.2013.0060

[45] Kwan, M., Jeemi, Z., Norman, R. & Dantas, J. A. R. Professional Interpreter Services and the Impact on Hospital Care Outcomes: An Integrative Review of Literature. *Int. J. Environ. Res. Public. Health* **20**, 5165 (2023). 36982073 10.3390/ijerph20065165 PMC10048935

[46] Payvandi, L., Parsons, C., Bourgeois, F. C. & Hron, J. D. Inpatient Telehealth Experience of Patients With Limited English Proficiency: Cross-sectional Survey and Semistructured Interview Study. *JMIR Form. Res.* **6**, e34354 (2022). 35438641 10.2196/34354 PMC9066319

[47] Tan-McGrory, A., Schwamm, L. H., Kirwan, C., Betancourt, J. R. & Barreto, E. A. Addressing virtual care disparities for patients with limited English proficiency. *Am. J. Manag. Care* **28**, 36–40 (2022). 35049259 10.37765/ajmc.2022.88814

[48] Galloway, J. L. et al. Impact of an Artificial Intelligence-Based Solution on Clinicians’ Clinical Documentation Experience: Initial Findings Using Ambient Listening Technology. *J. Gen. Intern. Med.* **39**, 2625–2627 (2024). 38980463 10.1007/s11606-024-08924-2 PMC11436573

[49] Tougas, H. et al. The Use of Automated Machine Translation to Translate Figurative Language in a Clinical Setting: Analysis of a Convenience Sample of Patients Drawn From a Randomized Controlled Trial. *JMIR Ment. Health* **9**, e39556 (2022). 36066959 10.2196/39556 PMC9490520

[50] Succop, B. et al. Automated translation accurately translates recorded pediatric neurosurgery clinic conversations between Spanish and English. *Neurosurg. Rev.* **47**, 210 (2024). 38724863 10.1007/s10143-024-02441-w

[51] Yeboah, D., McDaniel, C. & Lion, K. C. Language Matters: Why We Should Reconsider the Term “Limited English Proficiency. *Hosp. Pediatr.* **13**, e11–e13 (2022). 10.1542/hpeds.2022-007014 36464981

[52] Bhandari, A. & Wagner, T. Self-Reported Utilization of Health Care Services: Improving Measurement and Accuracy. 10.1177/1077558705285298 (2006). 10.1177/1077558705285298 16595412

[53] Moher, D., Liberati, A., Tetzlaff, J., Altman, D. G. & Group, T. P. Preferred Reporting Items for Systematic Reviews and Meta-Analyses: The PRISMA Statement. *PLOS Med* **6**, e1000097 (2009). 21603045 PMC3090117

