# Supplementary material for: How limited english proficiency impacts patient engagement with telemedicine: a systematic review
Source: NPJ Digit Med. 2025 Nov 21;8:717. doi: 10.1038/s41746-025-02090-3 (PMC12638763; doi:10.1038/s41746-025-02090-3)
Supplement: Supplementary file 1 — Supplementary information [file 41746_2025_2090_MOESM1_ESM.pdf]

Supplementary Information File

How Limited English Proficiency Impacts Patient Engagement with Telemedicine: A Systematic Review

Andrea Huang [1, 2], John Geracitano [3], Melissa Coffel [2], Carl Seashore [4], Saif Khairat [2,3,5*]

[1] Gillings School of Global Public Health, University of North Carolina at Chapel Hill, Chapel Hill, North Carolina, USA

[2] School of Nursing, University of North Carolina at Chapel Hill, Chapel Hell, North Carolina, USA.

[3] Carolina Health Informatics Program, University of North Carolina at Chapel Hill, Chapel Hill, North Carolina, USA.

[4] General Pediatrics and Adolescent Medicine, School of Medicine, University of North Carolina at Chapel Hill, Chapel Hill, North Carolina, USA.

[5] Lineberger Comprehensive Cancer Center, University of North Carolina at Chapel Hill, Chapel Hill, North Carolina, USA.

* Corresponding author – Dr. Saif Khairat, Email: Saif@unc.edu, Phone: 919-843-5413, postal address - 428 Carrington Hall, Campus Box 7460, Chapel Hill, NC 27599

Supplementary Table 1. Database Search Strategies

Database	Search Query
PubMed	(low proficiency English[tiab] OR "low English proficien*" [tiab] OR limited proficiency English[tiab] OR "limited English Proficien*"[tiab] OR "non-native speaker*"[tiab] OR "nonnative speaker*"[tiab] OR "limited fluency" [tiab] OR "non-English language" [tiab] OR "English proficien*"[tiab] OR "language barrier*"[tiab] OR "non-fluent"[tiab] OR "nonfluent" [tiab] OR (fluen*[tiab] AND English[tiab])) AND ("Telemedicine" [Mesh] OR telemedicine[tiab] OR telehealth[tiab] OR "virtual medicine" [tiab] OR "virtual care"[tiab] OR eHealth[tiab] OR telecare[tiab] OR tele-care[tiab] OR emedicine[tiab] OR "digital health"[tiab] OR "remote care"[tiab] OR "remote health care"[tiab] OR "remote consult*"[tiab] OR "virtual visit*"[tiab] OR "teleconsult*"[tiab])
Scopus	(TITLE-ABS("low proficiency English") OR TITLE-ABS("low English proficien*") OR TITLE-ABS("limited proficiency English") OR TITLE-ABS("limited English Proficien*") OR TITLE-ABS("non-native speaker*") OR TITLE-ABS("nonnative speaker*") OR TITLE-ABS("limited fluency") OR TITLE-ABS("non-English language") OR TITLE-ABS("English proficien*") OR TITLE-ABS("language barrier*") OR TITLE-ABS(non-fluent) OR TITLE-ABS(nonfluent) OR (TITLE-ABS(fluen*) AND TITLE-ABS(English))) AND (INDEXTERMS(Telemedicine) OR TITLE-ABS(telemedicine) OR TITLE-ABS(telehealth) OR TITLE-ABS("virtual medicine") OR TITLE-ABS("virtual care") OR TITLE-ABS(eHealth) OR TITLE-ABS(telecare) OR TITLE-ABS(tele-care) OR TITLE-ABS(emedicine) OR TITLE-ABS("digital health") OR TITLE-ABS("remote care") OR TITLE-ABS("remote health care") OR TITLE-ABS("remote consult*") OR TITLE-ABS("virtual visit*") OR TITLE-ABS(teleconsult*))
CINAHL	((TI "low proficiency English" OR AB "low proficiency English") OR (TI "low English proficien*" OR AB "low English proficien*") OR (TI "limited proficiency English" OR AB "limited proficiency English") OR (TI "limited English Proficien*" OR AB "limited English Proficien*") OR (TI "non-native speaker*" OR AB "non-native speaker*") OR (TI "nonnative speaker*" OR AB "nonnative speaker*") OR (TI "limited fluency" OR AB "limited fluency") OR (TI "non-English language" OR AB "non-English language") OR (TI "English proficien*" OR AB "English proficien*") OR (TI "language barrier*" OR AB "language barrier*") OR (TI non-fluent OR AB non-fluent) OR (TI nonfluent OR AB nonfluent) OR ((TI fluen* OR AB fluen*) AND (TI English OR AB English))) AND ((MH Telemedicine+) OR (TI telemedicine OR AB telemedicine) OR (TI telehealth OR AB telehealth) OR (TI "virtual medicine" OR AB "virtual medicine") OR (TI "virtual care" OR AB "virtual care") OR (TI eHealth OR AB eHealth) OR (TI telecare OR AB telecare) OR (TI tele-care OR AB tele-care) OR (TI emedicine OR AB emedicine) OR (TI "digital health" OR AB "digital health") OR (TI "remote care" OR AB "remote care") OR (TI "remote health care" OR AB "remote health care") OR (TI "remote consult*" OR AB "remote consult*") OR (TI "virtual visit*" OR AB "virtual visit*") OR (TI teleconsult* OR AB teleconsult*))
Embase	('low proficiency English':ti,ab OR 'low English proficien*':ti,ab OR 'limited proficiency English':ti,ab OR 'limited English Proficien*':ti,ab OR 'non-native speaker*':ti,ab OR 'nonnative speaker*':ti,ab OR 'limited fluency':ti,ab OR 'non-

	English language':ti,ab OR 'English proficien*':ti,ab OR 'language barrier*':ti,ab OR non-fluent:ti,ab OR nonfluent:ti,ab OR (fluen*:ti,ab AND English:ti,ab)) AND (Telemedicine/exp OR telemedicine:ti,ab OR telehealth:ti,ab OR 'virtual medicine':ti,ab OR 'virtual care':ti,ab OR eHealth:ti,ab OR telecare:ti,ab OR tele-care:ti,ab OR emedicine:ti,ab OR 'digital health':ti,ab OR 'remote care':ti,ab OR 'remote health care':ti,ab OR 'remote consult*':ti,ab OR 'virtual visit*':ti,ab OR teleconsult*:ti,ab)

Note: The following search terms (in PubMed syntax) were added during the final search phase conducted on May 28, 2025: "digital health"[tiab] OR "remote care"[tiab] OR "remote health care"[tiab] OR "remote consult*"[tiab] OR "virtual visit*"[tiab] OR "teleconsult*"[tiab]

Supplementary Table 2. Articles Excluded and Reasons for Exclusion in Full-Text Screening Phase

No Full Text (abstract only, conference abstracts and posters)

1. Casillas A, Valdovinos C, Wang E, et al. Perspectives from leadership and frontline staff on telehealth transitions in the Los Angeles safety net during the COVID-19 pandemic and beyond. *Front Digit Health*. 2022;4:944860. doi:10.3389/fdgth.2022.944860
2. Jacobs D, Zhu D, Watts K. EVALUATING WHY PATIENTS SELECT TELEPHONE VISITS OVER VIDEO VISITS IN AN OUTPATIENT UROLOGY PRACTICE. *J Urol*. 2022;207(SUPPL 5):e425-e426. doi:10.1097/JU.0000000000002567.11
3. Lee MS, Kassamali B, Shah N, Lachance A, Nambudiri V. 281 Racial and language disparities in teledermatology visits for acne during the COVID-19 pandemic. *J Invest Dermatol*. 2021;141(5):S50. doi:10.1016/j.jid.2021.02.303
4. Lyles CR, Fields JD, Aulakh V, et al. LANGUAGE-SPECIFIC BARRIERS AND FACILITATORS TO TELEMEDICINE USE FACED BY SAFETY-NET HEALTH CARE SETTINGS DURING THE COVID-19 PANDEMIC. *J Gen Intern Med*. 2022;37((Lyles C.R.) Epidemiology and Biostatistics, University of California San Francisco, San Francisco, CA, United States):S154. doi:10.1007/s11606-022-07653-8
5. Neeman E, Lyon L, Sun H, et al. The future of tele-oncology: Trends and disparities in telehealth and secure message utilization in the COVID-19 era. *J Clin Oncol*. 2021;39(15 SUPPL). doi:10.1200/JCO.2021.39.15_suppl.1506
6. Rodriguez JA, Saadi A, Schwamm L, Bates DW, Samal L. Disparities in telehealth use among patients with limited english proficiency: California health interview survey, 2015-2018. *J Gen Intern Med*. 2020;35(SUPPL 1):S90-S91. doi:10.1007/s11606-020-05890-3
7. Rodriguez NJ, Okwara NC, Shen L, Jajoo K, Chan WW. Telemedicine In The Covid-19 Era: Impact On Disparities In Access To Ambulatory Care. *Gastroenterology*. 2021;160(6):S-29. doi:10.1016/S0016-5085(21)00817-9
8. Roy M, Fardeen T, Cabot A, et al. Association of telemedicine use with disparities in cancer distress screening for patients with limited English proficiency. *J Clin Oncol*. 2021;39(28 SUPPL). doi:10.1200/JCO.2020.39.28_suppl.147
9. Santana JCP, Storino A, Garland ME, Callery MP, Kent TS. SOCIODEMOGRAPHIC DETERMINANTS OF MISSED TELEHEALTH APPOINTMENTS IN HPB SURGERY. *Gastroenterology*. 2022;162(7):S-1305. doi:10.1016/S0016-5085(22)63813-7
10. Townsend MJ, Henson JB, Wegermann K, Muir AJ. S1692 Trends and Disparities in Hepatology Televisits at a Large Tertiary Care Center. *Off J Am Coll Gastroenterol ACG*. 2023;118(10S):S1264. doi:10.14309/01.ajg.0000956408.97683.d0

Wrong Study Design (qualitative-only studies, feasibility/pilot studies, editorials, conference abstracts, protocols)

11. Bharadwaj M, Langbein B, Labban M, Lipsitz SR, Licurse AM, Trinh QD. Patterns and Disparities in Telehealth Usage During the COVID-19 Pandemic Across Surgical Specialties. *Telemed J E-Health Off J Am Telemed Assoc*. 2024;30(3):866-873. doi:10.1089/tmj.2022.0332
12. Obregon E, Ortiz R, Wallis KE, Morgan S, Montoya-Williams D. Feasibility, Acceptability, and Health Outcomes Associated With Telehealth for Children in Families With Limited English Proficiency. *Acad Pediatr*. 2024;24(1):13-22. doi:10.1016/j.acap.2023.06.025
13. Romain CV, Trinidad S, Kotagal M. The Effect of Social Determinants of Health on Telemedicine Access During the COVID-19 Pandemic. *Pediatr Ann*. 2022;51(8):e311-e315. doi:10.3928/19382359-20220606-04
14. Sharma AE, Lisker S, Fields JD, et al. Language-Specific Challenges and Solutions for Equitable Telemedicine Implementation in the Primary Care Safety Net During COVID-19. *J Gen Intern Med*. 2023;38(14):3123-3133. doi:10.1007/s11606-023-08304-2
15. Tan-McGrory A, Schwamm LH, Kirwan C, Betancourt JR, Barreto EA. Addressing virtual care disparities for patients with limited English proficiency. *Am J Manag Care*. 2022;28(1):36-40. doi:10.37765/ajmc.2022.88814
16. Uscher-Pines L, Kapinos K, Rodriguez C, et al. Access challenges for patients with limited English proficiency: a secret-shopper study of in-person and telehealth behavioral health services in California safety-net clinics. *Health Aff Sch*. 2023;1(3):qxad033. doi:10.1093/haschl/qxad033

Wrong Outcomes: no LEP-stratified outcomes, no telemedicine utilization outcomes.

17. Bailey S, Pack A, Rusca P, et al. DISPARITIES IN TELEHEALTH ACCESS AND USE AMONG PATIENTS IN FEDERALLY QUALIFIED HEALTH CENTERS DURING THE COVID-19 PANDEMIC. *J Gen Intern Med*. 2023;38((Bailey S.; Pack A.; Huang W.; Wismer G.; Zuleta R.) General Internal Medicine, Northwestern University, Feinberg School of Medicine, Chicago, IL, United States):S241-S242. doi:10.1007/s11606-023-08226-z
18. Hart S, Campbell C, Divine H, et al. Telehealth diabetes services for non-English speaking patients. *J Am Pharm Assoc JAPhA*. 2022;62(4):1394-1399. doi:10.1016/j.japh.2022.03.009
19. Khoong EC, Butler BA, Mesina O, et al. Patient interest in and barriers to telemedicine video visits in a multilingual urban safety-net system. *J Am Med Inform Assoc*. 2021;28(2):349-353. doi:10.1093/jamia/ocaa234 Khoong EC, Butler BA, Mesina O, et al. Patient interest in and barriers to telemedicine video visits in a multilingual urban safety-net system. *J Am Med Inform Assoc*. 2021;28(2):349-353. doi:10.1093/jamia/ocaa234
20. Khoong EC, Rivadeneira NA, Hiatt RA, Sarkar U. The Use of Technology for Communicating With Clinicians or Seeking Health Information in a Multilingual Urban Cohort: Cross-Sectional Survey. *J Med Internet Res*. 2020;22(4):e16951. doi:10.2196/16951

21. Kim EK, Kidane J, Brodie S, Tuot DS, Sharon JD. Utility of telephone visits at an urban safety-net hospital during 2020: A retrospective review. *Laryngoscope Investig Otolaryngol*. 2022;7(5):1315-1321. doi:10.1002/lio2.875

Wrong Patient Population

22. DeCamp LR, Williams L, Palmer C, Gorman C, Olson C, Thompson DA. Mixed methods evaluation of pediatric telehealth equity for patients/families who communicate in languages other than English. *mHealth*. 2023;9:24. doi:10.21037/mhealth-22-43
23. Payvandi L, Parsons C, Bourgeois FC, Hron JD. Inpatient Telehealth Experience of Patients With Limited English Proficiency: Cross-sectional Survey and Semistructured Interview Study. *JMIR Form Res*. 2022;6(4):e34354. doi:10.2196/34354

Wrong Exposure

24. Hsueh L, Huang J, Millman AK, et al. Cross-Sectional Association of Patient Language and Patient-Provider Language Concordance with Video Telemedicine Use Among Patients with Limited English Proficiency. *J Gen Intern Med*. 2023;38(3):633-640. doi:10.1007/s11606-022-07887-6

Supplementary Table 3. Quality Rating Scheme for Studies and Other Evidence (modified from the Oxford Centre for Evidence-based Medicine for ratings of individual studies by JAMA)[16]

Quality Rating Scheme for Studies and Other Evidence	
1	Properly powered and conducted randomized clinical trial; systematic review with meta-analysis
2	Well-designed controlled trial without randomization; prospective comparative cohort trial
3	Case-control studies; retrospective cohort study
4	Case series with or without intervention; cross-sectional study
5	Opinion of respected authorities; case reports

Supplementary Table 4. PRISMA Checklist

Section and Topic	Item #	Checklist item	Location where item is reported (page #)
**TITLE**			
Title	1	Identify the report as a systematic review.	1
**ABSTRACT**			
Abstract	2	See the PRISMA 2020 for Abstracts checklist.	2
**INTRODUCTION**			
Rationale	3	Describe the rationale for the review in the context of existing knowledge.	3, 4
Objectives	4	Provide an explicit statement of the objective(s) or question(s) the review addresses.	3, 4
**METHODS**			
Eligibility criteria	5	Specify the inclusion and exclusion criteria for the review and how studies were grouped for the syntheses.	11, 12
Information sources	6	Specify all databases, registers, websites, organisations, reference lists and other sources searched or consulted to identify studies. Specify the date when each source was last searched or consulted.	11, 12
Search strategy	7	Present the full search strategies for all databases, registers and websites, including any filters and limits used.	11, 12<br>Supplementary Table 1
Selection process	8	Specify the methods used to decide whether a study met the inclusion criteria of the review, including how many reviewers screened each record and each report retrieved, whether they worked independently, and if applicable, details of automation tools used in the process.	11, 12
Data collection process	9	Specify the methods used to collect data from reports, including how many reviewers collected data from each report, whether they worked independently, any processes for obtaining or confirming data from study investigators, and if applicable, details of automation tools used in the process.	11, 12
Data items	10a	List and define all outcomes for which data were sought. Specify whether all results that were compatible with each outcome domain in each study were sought (e.g. for all measures, time points, analyses), and if not, the methods used to decide which results to collect.	11, 12
	10b	List and define all other variables for which data were sought (e.g. participant and intervention characteristics, funding sources). Describe any assumptions made about any missing or unclear information.	11, 12
Study risk of bias assessment	11	Specify the methods used to assess risk of bias in the included studies, including details of the tool(s) used, how many reviewers assessed each study and whether they worked independently, and if applicable, details of automation tools used in the process.	12, Table 2
Effect measures	12	Specify for each outcome the effect measure(s) (e.g. risk ratio, mean difference) used in the synthesis or presentation of results.	Table 1
Synthesis methods	13a	Describe the processes used to decide which studies were eligible for each synthesis (e.g. tabulating the study intervention characteristics and comparing against the planned groups for each synthesis (item #5)).	N/A
	13b	Describe any methods required to prepare the data for presentation or synthesis, such as handling of missing summary statistics, or data conversions.	N/A
	13c	Describe any methods used to tabulate or visually display results of individual studies and syntheses.	N/A
	13d	Describe any methods used to synthesize results and provide a rationale for the choice(s). If meta-analysis was performed, describe the model(s), method(s) to identify the presence and extent of statistical heterogeneity, and software package(s) used.	N/A
	13e	Describe any methods used to explore possible causes of heterogeneity among study results (e.g. subgroup analysis, meta-regression).	N/A

Section and Topic	Item #	Checklist item	Location where item is reported (page #)
	13f	Describe any sensitivity analyses conducted to assess robustness of the synthesized results.	N/A
Reporting bias assessment	14	Describe any methods used to assess risk of bias due to missing results in a synthesis (arising from reporting biases).	12, Table 2
Certainty assessment	15	Describe any methods used to assess certainty (or confidence) in the body of evidence for an outcome.	12, Table 2
**RESULTS**			
Study selection	16a	Describe the results of the search and selection process, from the number of records identified in the search to the number of studies included in the review, ideally using a flow diagram.	4, Figure 1
	16b	Cite studies that might appear to meet the inclusion criteria, but which were excluded, and explain why they were excluded.	4, Supplementary Table 2
Study characteristics	17	Cite each included study and present its characteristics.	4, 5, 6, 7, 8, Table 1
Risk of bias in studies	18	Present assessments of risk of bias for each included study.	Table 2
Results of individual studies	19	For all outcomes, present, for each study: (a) summary statistics for each group (where appropriate) and (b) an effect estimate and its precision (e.g. confidence/credible interval), ideally using structured tables or plots.	Table 1
Results of syntheses	20a	For each synthesis, briefly summarise the characteristics and risk of bias among contributing studies.	4, 5, 6, 7, 8, Table 1, Table 2
	20b	Present results of all statistical syntheses conducted. If meta-analysis was done, present for each the summary estimate and its precision (e.g. confidence/credible interval) and measures of statistical heterogeneity. If comparing groups, describe the direction of the effect.	4, 5, 6, 7, 8
	20c	Present results of all investigations of possible causes of heterogeneity among study results.	4, 5, 6, 7, 8, Table 1
	20d	Present results of all sensitivity analyses conducted to assess the robustness of the synthesized results.	N/A
Reporting biases	21	Present assessments of risk of bias due to missing results (arising from reporting biases) for each synthesis assessed.	N/A
Certainty of evidence	22	Present assessments of certainty (or confidence) in the body of evidence for each outcome assessed.	Table 1, Table 2
**DISCUSSION**			
Discussion	23a	Provide a general interpretation of the results in the context of other evidence.	8, 9, 10, 11
	23b	Discuss any limitations of the evidence included in the review.	11
	23c	Discuss any limitations of the review processes used.	10, 11
	23d	Discuss implications of the results for practice, policy, and future research.	8, 9, 10, 11
**OTHER INFORMATION**			

Section and Topic	Item #	Checklist item	Location where item is reported (page #)
Registration and protocol	24a	Provide registration information for the review, including register name and registration number, or state that the review was not registered.	13
	24b	Indicate where the review protocol can be accessed, or state that a protocol was not prepared.	13
	24c	Describe and explain any amendments to information provided at registration or in the protocol.	13
Support	25	Describe sources of financial or non-financial support for the review, and the role of the funders or sponsors in the review.	13
Competing interests	26	Declare any competing interests of review authors.	13
Availability of data, code and other materials	27	Report which of the following are publicly available and where they can be found: template data collection forms; data extracted from included studies; data used for all analyses; analytic code; any other materials used in the review.	13

From: Page MJ, McKenzie JE, Bossuyt PM, Boutron I, Hoffmann TC, Mulrow CD, et al. The PRISMA 2020 statement: an updated guideline for reporting systematic reviews. BMJ 2021;372:n71. doi: 10.1136/bmj.n71
